# Mosaic chromosomal alterations (mCAs) in individuals with monoclonal B-cell lymphocytosis (MBL)

**DOI:** 10.1038/s41408-024-01175-8

**Published:** 2024-11-06

**Authors:** Aswin Sekar, Rosalie Griffin, Sameer A. Parikh, Giulio Genovese, Dennis P. Robinson, Aaron D. Norman, Janet E. Olson, Kari G. Rabe, Mingma S. Hoel, Nicholas J. Boddicker, Paul J. Hampel, Neil E. Kay, James R. Cerhan, Esteban Braggio, Curtis A. Hanson, Celine M. Vachon, Tait D. Shanafelt, Benjamin L. Ebert, Susan L. Slager

**Affiliations:** 1 Department of Medical Oncology, Dana-Farber Cancer Institute, Boston, MA USA; 2 Division of Computational Biology, Mayo Clinic, Rochester, MN USA; 3 Division of Epidemiology, Mayo Clinic, Rochester, MN USA; 4 Division of Hematology, Mayo Clinic, Rochester, MN USA; 5 Department of Genetics, Harvard Medical School, Boston, MA USA; 6 Division of Clinical Trials and Biostatistics, Mayo Clinic, Rochester, MN USA; 7 Department of Immunology, Mayo Clinic, Rochester, MN USA; 8 Department of Hematology/Oncology, Mayo Clinic, Phoenix, AZ USA; 9 Department of Laboratory Medicine and Pathology, Division of Hematopathology, Mayo Clinic, Rochester, MN USA; 10 Department of Medicine, Division of Hematology, Stanford University, Stanford, CA USA; 11 Department of Medicine, Brigham and Women’s Hospital and Harvard Medical School, Boston, MA USA; 12 The Broad Institute of Massachusetts Institute of Technology and Harvard, Cambridge, MA USA; 13 Howard Hughes Medical Institute, Dana-Farber Cancer Institute, Boston, MA USA

**Keywords:** B-cell lymphoma, Cancer genetics, Haematopoietic stem cells, Cancer prevention

## Abstract

MBL is a precursor condition to chronic lymphocytic leukemia (CLL), characterized by monoclonal B-cells in blood. Mosaic chromosomal alterations (mCAs) are a form of clonal hematopoiesis that include gains, losses, and copy-neutral loss-of-heterozygosity of large DNA segments. Both MBL and mCAs have been found to increase the risk of CLL and lymphoid malignancies, and the aim of our study was to investigate how mCAs relate to MBL, which is currently unknown. We analyzed genetic, flow cytometric, and hematologic data from 4632 individuals from the Mayo Clinic Biobank and CLL Database. MBL was detected using flow cytometry and classified as high-count (HC) or low-count (LC) MBL based on clone size. mCAs were detected primarily from whole blood DNA using sensitive SNP-array-based analyses. mCAs commonly altered in CLL (deletion of 6q, 11q, 13q, 17p, and trisomy 12) were specific (>99%) to individuals with MBL and CLL. HC-MBL and LC-MBL individuals were 881-fold and 8-fold, respectively, more likely to harbor CLL-associated mCAs than those without MBL. The cell fraction bearing these mCAs typically exceeded the B-cell fraction, suggesting their origin prior to the B-cell lineage. Integrating genetic and blood count data enabled detecting HC-MBL with high specificity in a biobank sample. These results quantify the contribution of mCAs to MBL and could enable large studies of HC-MBL without the need for flow cytometric screening.

## Introduction

Monoclonal B-cell lymphocytosis (MBL) is a precursor state to chronic lymphocytic leukemia (CLL) [1–3] and is characterized by the presence of a monoclonal B-cell population in the peripheral blood of individuals without other features of a lymphoproliferative disorder [4, 5]. Individuals with a clonal B-cell count less than 500 cells/μL are considered to have low-count MBL (LC-MBL) while those with clonal B-cell count between 500 and 5000 cells/μL have high-count MBL (HC-MBL). MBL is also commonly sub-classified based on the immunophenotype of the cell surface markers, with CLL-like clones being more common than those with atypical or non-CLL immunophenotypes. The prevalence of MBL increases with age, with a frequency of 4% among those in their 40 s to more than 40% among those older than 90 years of age [6]. Individuals with HC-MBL have an increased risk of a range of adverse clinical outcomes, including progression to CLL requiring therapy [7, 8], increased risk of lymphoid malignancies [6], solid tumors [9], and serious infections [10]. Although the majority of individuals (95%) with MBL have LC-MBL [6], it is an understudied condition. We previously found that individuals with LC-MBL have increased risk of lymphoid malignancies [6] (albeit lower risk compared to individuals with HC-MBL), serious infections [11, 12], and melanoma [13].

Similar to MBL, clonal hematopoiesis (CH) is an age-related asymptomatic hematological precursor condition characterized by the clonal expansion of blood cells [14, 15]. Clonal hematopoiesis of indeterminate potential (CHIP) is defined by the presence of somatic mutations present at a variant allele frequency (VAF) of at least 2% in the blood cells of individuals without a hematologic malignancy. Initial studies of CHIP analyzed somatic mutations in the form of point mutations or short insertions/ deletions in genes that are drivers of hematologic malignancies and characterized it primarily as a risk factor for myeloid malignancies [14, 15]. Mosaic chromosomal alterations (mCAs) are another form of CH, which affect large segments of DNA and include gains, losses, and copy-number neutral loss-of heterozygosity events [16]. We recently classified mCAs into those that specifically increase the risk of lymphoid versus myeloid malignancies [17]. Lymphoid mCAs included events that are commonly seen among CLL patients, such as deletions of 11q, 13q, and 17p, and trisomy 12. In our analysis of the UK Biobank, individuals with a lymphoid mCA had an 11-fold increased risk of subsequent lymphoid malignancies overall, and a 69-fold increased risk of CLL specifically, compared to individuals without a lymphoid mCA [17]. However, in the absence of flow cytometric data, the possibility that these relationships could simply reflect the presence of MBL among individuals with lymphoid mCAs could not be addressed in large biobank studies. Herein, we sought to address this question in a large, prospectively collected, and well-annotated cohort of individuals with flow cytometric, hematologic, clinical, and genetic data.

## Methods

### Study populations

#### Mayo Clinic participants

This study was approved by the institutional review boards of Mayo Clinic and Olmsted Medical Center. All participants provided written informed consent and were from either the Mayo Clinic MBL Biobank [6, 11, 12] or the Mayo Clinic CLL Database [9, 10, 18–20]. Among participants in the Mayo Clinic MBL Biobank, individuals had no prior history of hematological cancer and were 40 years or older at the time of phlebotomy. MBL screening was conducted on peripheral blood mononuclear cells (PBMCs) using an eight-color flow cytometry assay capable of detecting clonal B-cell events to the 0.005% level [6, 11]. Since not all Biobank participants had a complete blood count within a year of flow cytometry screening, we classified individuals by the size of the clone relative to total B-cells: HC-MBL as clonal B-cell count ≥85% out of total B-cell count [2, 18] and LC-MBL otherwise. The Mayo Clinic CLL Database [9, 10, 18, 21] includes individuals seen in the Division of Hematology at Mayo Clinic, Rochester, MN, with HC-MBL, CLL, or small lymphocytic lymphoma (SLL). Fluorescence in situ hybridization (FISH) data were among the clinical characteristics collected at the time of diagnosis, and this enabled the evaluation of the accuracy of our mCA calls.

#### Mass General Brigham Biobank participants

Data for participants from the Mass General Brigham Biobank (MGBB) [22, 23], who are recruited from clinics through the MGB system, were accessed under an approved secondary use authorization protocol. Since the MGBB has not been screened for MBL, we identified participants with (1) SNP-array data (for identification of mCAs) generated by the MGBB and (2) peripheral blood flow cytometry testing as part of their clinical care. There were 116 such individuals of whom 12 had HC-MBL and 104 did not have MBL, and these individuals served as a replication cohort as further detailed in the Supplementary Methods.

### DNA genotyping of the Mayo Clinic participants

DNA was extracted from whole blood for most individuals (100% of individuals without MBL, 99.7% LC-MBL, 90% HC-MBL, 99% SLL, and 95% CLL) and from PBMCs for the remainder. DNA was extracted from the same blood draw as that used for flow cytometry screening among the participants in the Mayo Clinic Biobank, enabling assessment of the relationship between mCAs and MBL at the same time point. Genotyping was performed using the Infinium OmniExpress Array or the Infinium Global Screening Array. Extensive quality control was implemented as detailed elsewhere [20, 24]. The quality-controlled genotyping data was used to compute a CLL-polygenic risk score (CLL-PRS) from the weighted average of 41 SNPs previously associated with CLL [20, 24] and to estimate genetic ancestry using ADMIXTURE [25].

### mCA detection from SNP-array data

Genotype intensity data were used to call mCAs with the MoChA algorithm [16], which enables the detection of mCAs to a sensitivity of 0.5–1% cell fraction. Event boundaries were determined by resampling, and log R ratio data was incorporated to classify the detected mCA as loss, copy-number neutral loss-of heterozygosity (LOH), or gain. Likely false positive calls were removed based on sample call rate, high B-allele frequency, low phase quality, and the likelihood of germline events [16].

### Classification of mCAs into canonical CLL-associated mCAs, CLL-driver mCAs, and lymphoid mCAs

#### Canonical CLL-associated mCAs

Chromosomal abnormalities typically tested on CLL clinical FISH panels [26] were classified as canonical CLL-associated mCAs and included the following: del 6q, del 11q, trisomy 12, del 13q, and del 17p and copy-number neutral LOH events at 13q/*MIR16-1*.

#### CLL-driver mCAs

CLL-driver mCAs were defined as mCAs that were either a canonical CLL-associated mCA as defined above and/or those that fully contained a chromosomal abnormality from two recent, large-scale genomic sequencing studies of CLL [27, 28], which collectively identified 179 unique candidate driver chromosomal abnormalities.

#### Lymphoid mCAs

Lymphoid mCAs were identified based on a pre-determined list of mCAs found in our earlier work [17] to be specifically associated with prevalent lymphoid malignancies in the UK Biobank.

### Statistical analyses of mCA association with MBL, CLL, and SLL

We evaluated associations between the presence of each mCA category as a predictor variable and the phenotypes being compared (e.g., HC-MBL vs. LC-MBL) as the outcome variable. We used logistic regression to estimate odds ratios (OR) and 95% confidence intervals (CI). Regression models were adjusted for age, sex, ancestry, DNA source, mCA calling batch, and array type (Supplementary Methods). To evaluate the ability to identify individuals with HC-MBL in biobank data, we compared HC-MBL against the combined group of individuals with LC-MBL and individuals without MBL. Regression models incorporating age, sex, mCA, CLL-PRS, and absolute lymphocyte count (ALC) data were generated. The performance of prediction models was estimated using ten-fold cross-validation. Analyses were performed and figures were generated using Rstudio version 2023.12.1 + 402.

Additional information regarding the study populations and methods described above is provided in the Supplementary Material.

## Results

### Study participants

We analyzed a total of 4632 individuals from the Mayo Clinic MBL Biobank [6] or the Mayo Clinic CLL Database [19–21] with available SNP-array data and clonal B-cell count data. Individuals screened for MBL from the Mayo Clinic MBL Biobank included 2971 individuals without MBL, 728 with LC-MBL, and 65 with HC-MBL. Participants from the Mayo Clinic CLL Database included 267 individuals with HC-MBL, 497 with CLL, and 104 with SLL. In the full cohort, the median age ranged from 65 years in individuals without MBL to 72 in individuals with LC-MBL (Supplementary Fig. 1A). The proportion of males ranged from 40.1% among those without MBL to 71.7% among CLL patients (Supplementary Fig. 1B). CLL-like MBL, defined by CD5+, CD20 dim, CD23+, was the most common immunophenotype (Supplementary Fig. 1C). The vast majority (>98%) of individuals were of European ancestry (Supplementary Fig. 1D). Distribution of absolute lymphocyte count is shown in Supplementary Fig. 1E.

### Relationship of mCAs with MBL, CLL, and SLL

We detected mCAs in the form of copy-number losses, gains, and copy-number neutral loss-of-heterozygosity (CNN-LOH) events in blood DNA from SNP-array data, using an approach that enables highly sensitive detection of mCAs down to a cell fraction of 0.5–1% [16]. Among the 4632 individuals in our analysis, we identified 2721 mCAs (1213 individuals, 26.1% with at least one mCA), of which 2322 mCAs were autosomal (912 individuals, 19.6% with at least one autosomal mCA), 325 were loss-of-Y (LoY; 15.0% among 2170 males), 41 were loss-of-X (LoX; 1.7% among 2462 females), and 33 were mCAs involving segments of the sex chromosomes (Supplementary Fig. 2). In total, these 2721 mCAs comprised 202 unique mCA events (defined by the type of mCA and the chromosome or chromosome-arm involved; Supplementary Table 1). We evaluated the sensitivity and specificity of mCA detection against data from clinical FISH assays available for individuals from the Mayo Clinic CLL Database and found the results to be highly specific (>96% per locus) but with variable sensitivity (ranging from 60% for 13q to 94% for 11q deletions) (Supplementary Table 2). Manual review of probe intensity data from SNP arrays recovered additional events and identified CNN-LOH events among those with del 13q detected by FISH (Supplementary Methods).

**Fig. 1:**
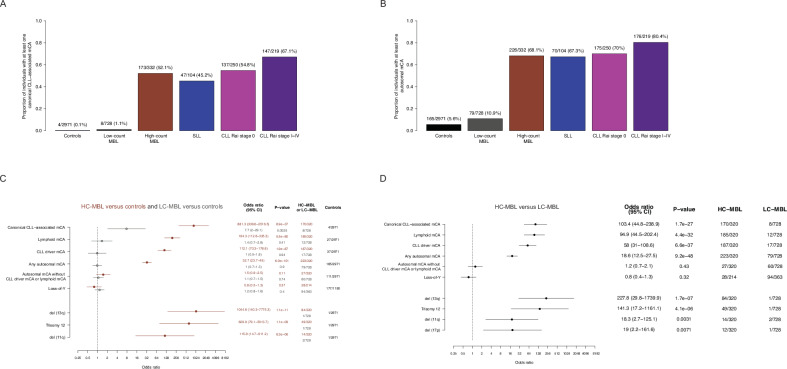
Relationship of mosaic chromosomal alterations (mCAs) with MBL. Proportion of individuals with at least one canonical CLL-associated mCA (**A**) and autosomal mCA (**B**). Association of different categories of mCAs (defined below) with HC-MBL vs. controls in brown and LC-MBL vs. controls in dark gray (**C**) and HC-MBL vs. LC-MBL (**D**). **‘**Controls’ refers to individuals in whom flow cytometry screening did not identify a B-cell clone in peripheral blood mononuclear cells. Covariates in (**C**) and (**D**) included age, sex, European ancestry, SNP-array type, source of DNA (whole blood or PBMC), and batch effects. del 17p is not shown in (**C**) as it was not found in any of the controls, and association statistics for del 11q, trisomy 12, and del 13q are not displayed for LC-MBL given their rarity in both this category and controls. Canonical CLL-associated mCA: del 6q, del 11q, trisomy 12, del 13q, del 17p, and copy-number neutral loss-of heterozygosity at 13q/ *MIR16-1* at 13q/ *MIR16-1*. CLL-driver mCA: includes canonical CLL-associated mCAs as well as those that were among 179 candidate drivers of CLL identified in two large genomic studies of CLL [27, 28]. Lymphoid mCA: mCAs whose frequency was specifically enriched in individuals with lymphoid malignancies in comparison to myeloid malignancies [17]. Autosomal mCA without CLL-driver mCA or lymphoid mCA: autosomal mCAs in individuals who have neither a CLL-driver mCA nor a lymphoid mCA.

We first investigated the relationship of MBL with a set of canonical CLL-associated mCAs – those typically tested on clinical CLL FISH panels, including deletions of 6q, 11q, 13q, 17p, trisomy 12, in addition to CNN-LOH of 13q. These canonical CLL-associated mCAs were common in HC-MBL (52.1%, 173/332), but rare among individuals with LC-MBL (1.1%, 8/728) or without MBL (0.1%, 4/2,971) (Fig. 1A). Of the four individuals without MBL carrying a canonical CLL-associated mCA, one subsequently developed LC-MBL when evaluated with flow cytometry at a second time point (the other three did not have a follow-up sample available). Thus, we found that canonical CLL-associated mCAs were highly specific to HC-MBL compared those without MBL (specificity 99.9%, 2967/2971). We sought to evaluate the reproducibility of this finding in an independent cohort, the Mass General Brigham Biobank. We identified 116 individuals with SNP-array data, with clinical flow cytometry assessment, and without a blood cancer diagnosis. Of these 116 individuals, 12 had HC-MBL, and 104 had no MBL. Of the 12 individuals with HC-MBL, 8 had canonical CLL-associated mCAs detected (66%, 8/12). Of the 104 individuals without MBL, only one had a canonical CLL-associated mCA (1%, 1/104). These results are consistent with those in the Mayo cohort. Taken together, the specificity of canonical CLL-associated mCAs for HC-MBL suggests the thousands of individuals in biobanks who harbor canonical CLL-associated mCAs [16, 17, 29] may have HC-MBL. Alternatively, these individuals could have undiagnosed CLL/SLL as canonical CLL-associated mCAs were also common among individuals with CLL/SLL (45.2%–67.1%, Fig. 1A) in the Mayo cohort, although the prevalence of diagnosed CLL/SLL is lower than that of HC-MBL in the general population.

Next, we evaluated a larger set of 179 CLL-driver mCAs identified in recent CLL genomic studies [27, 28]. Similar to canonical CLL-associated mCAs, these CLL-driver mCAs were common in HC-MBL and CLL/SLL (observed in 55.8% to 74.4% of individuals) and remained rare among individuals with LC-MBL (2.3%) and those without MBL (1.2%) (Supplementary Fig. 3).

Given the high specificity of canonical CLL-associated mCAs and CLL-driver mCAs for the presence of circulating B-cell clones, we examined whether their presence was restricted primarily to individuals with CLL-like MBL. We did detect canonical CLL-associated mCAs and CLL-driver mCAs across the different MBL immunophenotypes (Supplementary Fig. 4A, B). However, because the majority of individuals with MBL had CLL-like MBL (Supplementary Fig. 1C), we were not statistically powered to quantitatively assess differences of mCA frequencies across the MBL immunophenotypes. For subsequent analyses, we thus jointly analyzed the different immunophenotypes comprising MBL.

Given that MBL predisposes to lymphoid malignancies beyond CLL [6], we also investigated lymphoma-associated mCAs (lymphoid mCAs) defined in our previous work [17]. Lymphoid mCAs had a similar distribution and specificity across the spectrum of B-cell clonality as CLL-driver mCAs (Supplementary Fig. 5A). When analyzing the proportion of individuals who had a lymphoid mCA without any known chromosomal abnormalities that are candidate drivers of CLL [27, 28], the only category with an appreciable frequency of lymphoid mCAs was SLL (6/104; 5.6%, Supplementary Fig. 5B), a condition defined primarily by lymph node, rather than blood, involvement with CLL-like B-cell clones. The proportion of individuals who had autosomal mCAs without CLL-driver or lymphoid mCAs was similar across the B-cell clonality spectrum (Supplementary Fig. 5C).

We estimated the effect of carrying an mCA on MBL status in analyses adjusting for covariates. Compared to individuals without MBL, the presence of a canonical CLL-associated mCA was highly associated with HC-MBL (OR = 881, 95% CI: 308–2516, *p* = 8.9e-37, Fig. 1C). CLL-driver mCAs (OR = 112, 95% CI: 70–179, *p* = 1.9e-87) and lymphoid mCAs (OR = 194, 95% CI: 112.6–335.3, *p* = 5.5e-80) were significantly enriched in individuals with HC-MBL compared to individuals without MBL. The presence of any autosomal mCA was associated with HC-MBL (OR = 33, 95% CI: 24–45, *p* = 6.9e-101), but when CLL-driver and lymphoid mCAs were removed, the presence of a remaining autosomal mCA was not significantly associated. In LC-MBL, only canonical CLL-associated mCAs were significantly enriched (OR = 7.7, 95% CI: 2.0–29.1, *p* = 0.003) compared to individuals without MBL (Fig. 1C).

We next evaluated the differences in mCA prevalence in HC-MBL compared to LC-MBL. Canonical CLL-associated mCAs (OR = 103, 95% CI: 45–239, *p* = 1.7e-27), CLL-driver mCAs (OR = 58, 95% CI: 31–109, *p* = 6.6e-37), lymphoid mCAs (OR = 95, 95% CI: 45–202, *p* = 4.4e-32), and any autosomal mCA (OR = 19, 95% CI: 13–28, *p* = 9.2e-48) were significantly more common in individuals with HC-MBL compared to LC-MBL. The presence of autosomal mCAs in individuals without a CLL-driver mCA or lymphoid mCA did not significantly differ between HC-MBL and LC-MBL (Fig. 1D).

**Fig. 2:**
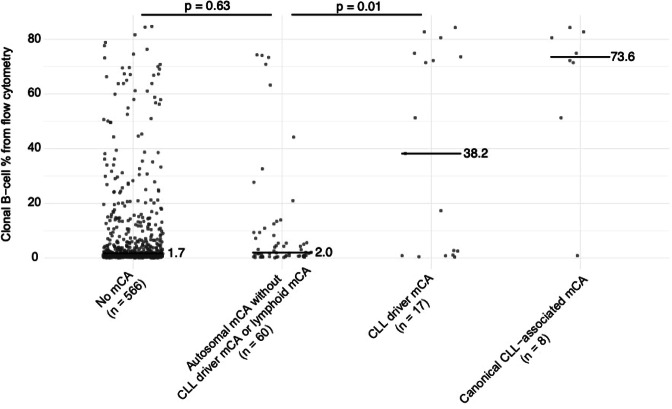
Evaluation of sensitivity to detect mCAs in blood DNA as an explanation for lower frequency of mCAs within low-count MBL. Clonal B-cell % from flow cytometry, which is clonal B-cells as a percentage of total B-cells, is shown for individuals with low-count MBL as a function of the type of mCAs present in each individual. Black horizontal bars and adjacent text indicate median values and *p*-values comparing clone size distribution are from a two-sided Mann–Whitney test.

We questioned whether our observed differences in mCA frequency between HC-MBL and LC-MBL could be due to lower sensitivity to detect mCAs given the smaller size of the B-cell clone in LC-MBL. If this were the case, we would also see a higher distribution of B-cell clone size (determined by flow cytometry) among LC-MBL individuals with autosomal mCAs that are not known to drive CLL or lymphoid malignancies than that among LC-MBL individuals without any mCA detected. However, this was not the case as these two distributions were statistically indistinguishable (*p* = 0.63 by Mann–Whitney test, Fig. 2). In contrast, the B-cell clone size distribution was higher among the few LC-MBL individuals with a CLL-driver mCA compared with those who only had autosomal mCAs that were not drivers of CLL or other lymphoid malignancies (*p* = 0.01, Fig. 2). These results suggest that the lower frequency of mCAs among individuals with LC-MBL is unlikely to be driven entirely by mCA detection sensitivity.

mCAs were significantly enriched in CLL/SLL cases compared to individuals without MBL (Fig. 1A, B, Supplementary Fig. 6A, B) and compared to individuals with HC-MBL with respect to their presence, number, and cell fraction (Supplementary Fig. 6C–E), though this degree of enrichment in CLL compared to HC-MBL was modest in contrast to that seen in HC-MBL versus LC-MBL and individuals without MBL (Fig. 1C, D). Compared to HC-MBL, the degree of enrichment in del 11q and del 17p was greater in CLL Rai stage I–IV than in CLL Rai stage 0. Otherwise, the frequency of different categories of mCAs was comparable across CLL Rai stage 0 and stages I–IV, consistent with mCAs being initiating events in CLL pathogenesis. When comparing CLL with SLL cases (Supplementary Fig. 6F), there was a trend toward trisomy 12 events being more common in SLL (as observed in smaller studies [30]) and deletion 11q being more common in CLL. The cell fraction of CLL-driver mCAs and the number of autosomal mCAs was higher in HC-MBL, SLL, and CLL, in comparison to LC-MBL and individuals without MBL (Supplementary Fig. 7A–D), consistent with these states being defined, in part, based on B-cell clone sizes [31].

### Inference of mCA blood cell lineage

**Fig. 3:**
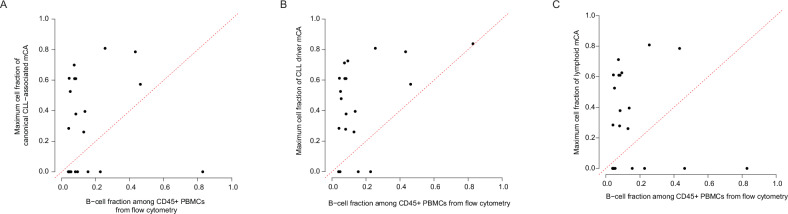
Inference of lineage distribution of mCAs by comparison of mCA cell fraction against B-cell fraction. Data are shown for canonical CLL-associated mCAs (**A**), CLL-driver mCAs (**B**), and lymphoid mCAs (**C**), the classification of which is detailed in the “Methods” section. Each point represents a single individual with HC-MBL from the Mayo Clinic MBL Biobank. For all individuals shown, flow cytometry was performed on frozen peripheral blood mononuclear cells (PBMCs) and DNA was extracted from PBMCs sampled on the same day as for flow cytometry. Data points with mCA cell fraction of 0 indicate individuals in whom the specified mCA type was not detected. Data points above the dashed red line indicate individuals in whom the fraction of cells containing a canonical CLL-associated mCA exceeds the B-cell fraction, suggesting the presence of the mCA beyond the B-cell lineage and origin prior to B-cell lineage commitment.

Given the high specificity of canonical CLL-associated mCAs for HC-MBL and CLL/SLL, we asked whether this finding reflects the lineage restriction of these mCAs to the B-cells. Mutations driving myeloid clonal hematopoiesis are thought to arise in hematopoietic stem or progenitor cells based on their presence across the hematopoietic differentiation tree [32], persistence over many years [33], and direct detection in hematopoietic stem cells (HSCs) [34]. Lymphoid clonal hematopoiesis [17], on the other hand, could originate either in hematopoietic stem or progenitor cells or after commitment to the B-cell lineage, given that some B-cells can persist long-term with proliferative and mutagenic capacity [35]. To test if canonical CLL-associated mCAs were restricted to the B-cell lineage, we compared an estimate of the fraction of cells harboring the mCA against the fraction of B-cells detected through flow cytometry. We expected the mCA cell fraction would be no greater than the flow cytometry B-cell fraction if these mCAs were restricted to the B-cell lineage. There were 22 individuals with HC-MBL whose DNA was extracted on the same sample source (peripheral blood mononuclear cells) as used for flow cytometry. Among the 12 HC-MBL individuals with a canonical CLL-associated mCA, all 12 individuals had a higher mCA cell fraction than the B-cell fraction (Fig. 3A); in the remaining 10 individuals, no canonical CLL-associated mCAs were detected. Similarly, 15 out of the 16 HC-MBL individuals with a CLL-driver mCA had higher mCA cell fractions than the B-cell fraction (Fig. 3B). All 13 individuals with a lymphoid mCA had higher mCA cell fraction than the B-cell fraction (Fig. 3C). Even among individuals for whom the source of DNA for mCA analysis was whole blood (rather than PBMCs, which were used for flow cytometry analysis in the MBL cohort), the mCA cell fraction exceeded the B-cell fraction in a considerable proportion of individuals (Supplementary Fig. 8A, C). These data support a cell-of-origin for these mCAs prior to commitment to the B-cell lineage in at least a subset of individuals. However, the less likely possibility of the same mCA arising independently in multiple lineages is not definitively ruled out by these data. Taken together, these results suggest that although CLL-associated mCAs are highly specific for the presence of circulating B-cell clones, their presence is not necessarily restricted to the B-cell lineage.

### Prediction of HC-MBL status using genetic and hematologic data

**Fig. 4:**
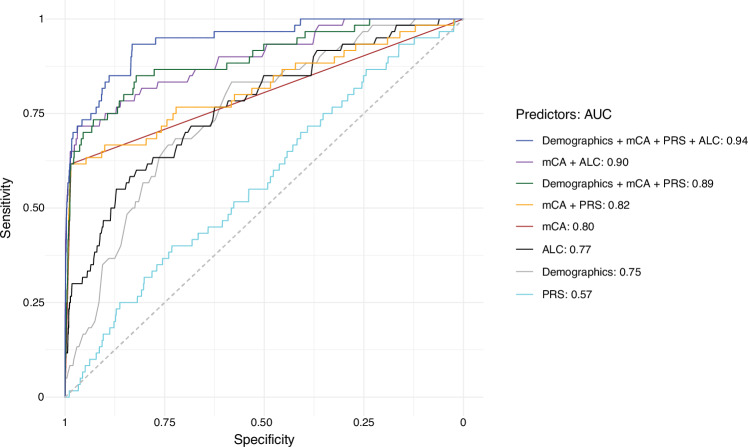
Test characteristics for distinguishing individuals with HC-MBL from controls and those with LC-MBL. The mCA parameter modeled here is the presence of at least one CLL-driver mCA. Demographics refers to age and sex. ALC absolute lymphocyte count. PRS polygenic risk score associated with CLL. This analysis is based on individuals with available data across all the predictors among HC-MBL cases in the Mayo Clinic MBL Biobank (*n* = 60), controls (*n* = 2740), and LC-MBL (*n* = 669).

Based on the strong relationships of HC-MBL with mCAs that we found above, we hypothesized that mCAs could serve as surrogates for HC-MBL status. While CLL-driver mCAs had a high specificity (98.5%, 3645/3699) for HC-MBL in comparison to the combined group of individuals with LC-MBL and those without MBL, they had a lower sensitivity of 57.2% (190/332, Supplementary Fig. 3). However, we reasoned that incorporating other types of data readily available in large biobanks [36] could further empower this detection. Our prior work [24] found that a CLL-polygenic risk score (CLL-PRS), calculated as the weighted average of the number of risk alleles of 41 SNPs identified from genome-wide association studies of CLL, was higher among individuals with MBL compared to those without. We developed regression models integrating the presence of demographic information (age and sex), mCAs, CLL-PRS, and absolute lymphocyte count (ALC) to evaluate their ability to distinguish individuals with HC-MBL from the combined group of individuals without MBL and LC-MBL. In ten-fold cross-validation analyses, ALC alone had an area under the receiver operating characteristic curve (AUC) of 0.77, the presence of at least one CLL-driver mCA alone had an AUC of 0.80, and the combination of demographics, CLL-driver mCA presence, and CLL-PRS had an AUC of 0.89. Incorporating demographics, CLL-driver mCA presence, CLL-PRS, and ALC increased the AUC to 0.94 (Fig. 4, Supplementary Fig. 9A–C). When comparing HC-MBL with Rai Stage 0 CLL, only ALC distinguished the two groups, as expected, and mCAs did not add additional information beyond ALC (Supplementary Fig. 9D).

## Discussion

We investigated the relationship between mCAs detected in whole blood DNA and MBL in a large, prospectively collected, well-annotated cohort of individuals with flow cytometric, hematologic, and clinical data. We found that CLL-associated mCAs were significantly more common in individuals with HC-MBL and rare in individuals without MBL and those with LC-MBL. Although CLL-associated mCAs were highly specific for the presence of circulating B-cell clones, their presence did not necessarily appear to be restricted to the B-cell lineage. Our results indicate that it may be possible to identify individuals with HC-MBL based on genetic and blood count data already available in biobanks, without the need for flow cytometric screening.

The current definitions of LC-MBL, HC-MBL, and CLL are based on quantitative cutoffs that would suggest that these states occupy different positions in the same continuum of B-cell clonality [37]. However, our results add to findings that reveal the ways in which these states may be biologically and qualitatively demarcated. The frequency of mCAs was similar in HC-MBL and CLL/SLL, but markedly lower in LC-MBL. These findings are consistent with prior studies that show similarities in the patterns of chromosomal alterations detected by FISH [38, 39], *IGHV* gene usage [7, 37], stereotyped B-cell receptors [40], and mutation profiles [41, 42] between HC-MBL and early stage CLL/SLL, as well as different IGHV usage for LC-MBL relative to CLL [43, 44]. We found that sensitivity to detect mCAs in whole blood is unlikely to fully explain their relatively low frequency among individuals with LC-MBL. However, prior studies using FISH on purified B-cells [44, 45] have reported a higher frequency of CLL-associated chromosomal abnormalities in LC-MBL. A limitation of our analyses is that, while the specificity of mCA detection was high (>96%) in comparison to data from clinical FISH assays, the sensitivity was variable across specific canonical CLL loci, with lower sensitivity for del 13q and trisomy 12. The implications of this limitation include that our findings may underestimate (i) the strength of association of canonical CLL-associated mCAs with HC-MBL compared to those without MBL; and (ii) the frequency of these mCAs among individuals with LC-MBL. Whether the small subset of individuals with LC-MBL that did have detectable driver mCAs and larger B-cell clone sizes are at higher risk for malignancy, or in transit to HC-MBL or CLL, remains to be determined. Addressing this question could have significant implications with respect to risk stratification of individuals with LC-MBL, a condition that is otherwise quite common in the general population [6] and for which there is currently a lack of recommendations regarding which, if any, LC-MBL individuals should be monitored clinically.

Although CLL-associated mCAs were highly specific for the presence of circulating B-cell clones, they did not appear to be restricted to the B-cell lineage. Given that the cell fraction estimates, both as they pertain to mCAs as well as to the B-cell fraction from flow cytometry, are subject to imprecision, we interpret our calculation of the proportion of HC-MBL individuals in whom the mCA cell fraction exceeded the B-cell fraction with caution. Indeed, experimental validation of our results in future studies would lend further support to the presence of CLL-associated mCAs beyond the B-cell lineage. However, while our analyses to infer blood cell lineage distribution of mCAs were indirect, direct experimental evidence from prior studies that have detected such chromosomal abnormalities in HSCs [46, 47] in CLL patients does support this finding. These results suggest that the high specificity of CLL-associated mCAs for B-cell clones is not merely a reflection of their origin in the B-cell lineage.

Currently, MBL is typically identified using flow cytometry. As this requires viable peripheral blood cells, the largest study of MBL to date is our MBL cohort which has 1712 MBL cases out of 10,139 individuals screened [6]. While flow cytometry data are not usually available in large-scale biobanks, genetic and hematologic data are abundant across many biobanks. The high specificity of CLL-associated mCAs for the presence of B-cell clones suggests that there may be large numbers of individuals in biobanks [16, 17, 29, 48] who carry such mCAs without a known blood cancer diagnosis and may have undiagnosed HC-MBL or CLL/SLL. Thus, these biobanks may have tremendous potential to provide insights regarding the clinical sequela of HC-MBL.

The capacity of mCAs to potentially serve as surrogates for HC-MBL fits with several earlier observations. First, strong parallels exist between epidemiologic observations in individuals with HC-MBL and those with mCAs. As with MBL, mCAs increase the risk of CLL [16, 17], infections [29], and non-hematologic cancers [29]. Second, mCAs tend to be enriched in individuals with high lymphocyte counts [16], consistent with mCAs being drivers or markers of lymphoid clonal growth. Third, in individuals who go on to develop CLL, mCAs are enriched among the somatic mutations found prior to diagnosis in comparison to point mutations in driver genes [17]. Fourth, large-scale sequencing studies of CLL patients have identified chromosomal alterations as early, initiating events in CLL pathogenesis [49].

The ability to identify individuals with HC-MBL using genetic and hematologic data would provide a powerful opportunity to study it at unprecedented scales. Biobank-scale studies of clonal hematopoiesis, which is detectable directly from genetic analyses of blood DNA, have enabled well-powered investigations into its inherited susceptibility [29, 50, 51] and phenotypic consequences [52–54] based on analyses of hundreds of thousands of individuals. HC-MBL is already known to predispose to lymphoid malignancies [6], infections [10], and non-hematologic cancers [9], and the ability to conduct similar large-scale studies could yield more comprehensive insights into its causes and the range of clinical outcomes to which HC-MBL predisposes.

Overall, our findings (i) quantify the contribution of mCAs to MBL, with a high specificity of CLL-associated mCAs for HC-MBL and CLL/SLL; (ii) support their presence beyond the B-cell lineage; and (iii) reveal the potential for mCAs to inform detection of HC-MBL in the research setting. Whether mCAs could help to stratify individuals with HC-MBL at a higher risk of developing lymphoid malignancies will require investigation in larger cohorts. The ability to identify such individuals could lay the foundation for ultimately developing and targeting interventions to potentially prevent progression to frank malignancies.

## Supplementary information

Supplementary_text_and_supplementary_figures

Supplementary_table1

Supplementary_table2

## Data Availability

For original data, please contact Slager.Susan@mayo.edu.
