# Supplementary material for: Mosaic chromosomal alterations (mCAs) in individuals with monoclonal B-cell lymphocytosis (MBL)
Source: Blood Cancer J. 2024 Nov 6;14(1):193. doi: 10.1038/s41408-024-01175-8 (PMC11541990; doi:10.1038/s41408-024-01175-8)
Supplement: Supplementary file 1 — Supplementary_text_and_supplementary_figures [file 41408_2024_1175_MOESM1_ESM.docx]

The Supplemental Methods section below contains additional details beyond those provided in the Methods section of the main text manuscript. The content that is new in the supplemental section is italicized. Supplemental Figures are included following the Supplemental Methods.

**Supplemental Methods**

Mayo Clinic participants*:* *The MBL Biobank consists of participants identified from the Mayo Clinic Biobank, a large-scale biorepository with biological samples and health information from 56,959 individuals who were recruited from primary care clinics between 2009-2016. A random sample of 10% of the 56,959 Mayo Clinic Biobank participants were selected to have peripheral blood mononuclear cells (PBMCs) banked, and thus were available to screen for MBL. Starting in 2017, we assembled a second cohort for MBL screening from the 90% of individuals participating in the Mayo Clinic Biobank who were not randomized to have stored PBMCs, resided in the 27 counties surrounding Mayo Clinic, Rochester, MN,* were 40 years or older, and had no prior history of hematologic malignancy. Since not all biobank participants had a complete blood count obtained within a year of research samples, we classified individuals by the size of the clone relative to total B-cells: HC-MBL as percent clonal B-cell count ≥85% out of total B-cell count and LC-MBL otherwise. *We justify this classification based on our previous finding(1, 2) that a cutoff of 85% clonal B-cells had a sensitivity and specificity of > 90% for HC-MBL.*

The Mayo Clinic CLL Database(3-5) includes individuals clinically seen in the Division of Hematology, Mayo Clinic, Rochester, MN, with HC-MBL, CLL, or SLL (small lymphocytic leukemia)*. All diagnoses were confirmed by a Mayo hematopathologist based on the 2008 International Workshop CLL criteria. Clinical characteristics were collected at the time of diagnosis, including genetic abnormalities detectable by fluorescence in situ hybridization (FISH) (e.g., trisomy 12 and deletions at 11q, 13q, and 17p*).

Mass General Brigham Biobank participants*:* Data for participants from the Mass General Brigham Biobank (MGBB)(6, 7), who are recruited from clinics through the MGB system, were accessed under an approved secondary use authorization protocol. Since the MGBB has not been screened for MBL, we identified participants with SNP array data (for identification of mCAs) and peripheral blood flow cytometry testing as part of their clinical care. *Of the 27,778 MGBB participants who SNP array data we had analyzed with MoChA(8) to call mCAs, we identified 5,340 individuals who had undergone multi-color flow cytometry testing for a panel of lymphoma markers as part of their clinical care. We excluded individuals (i) with a current or previous diagnoses of lymphoid malignancies, (ii) receiving anti-CD20 antibody therapies, (iii) with flow cytometry performed for monitoring after organ transplant, or (iv) insufficient B-cells for confident evaluation of clonality. Of the remaining 116 individuals, 12 had HC-MBL and 104 were negative for MBL*. *We note the following implications of having used this set of individuals as a replication cohort. Given that these were individuals who needed to have flow cytometry sent as part of their clinical care, they likely represent a sample of individuals who may not be as healthy as a general biobank sample in which flow cytometry was used for screening rather than for clinical diagnosis. Furthermore, many of these individuals are likely to have had cytopenias or cytoses as the indication for having had flow cytometry sent. However, this makes the reproducibility and generalizability of our finding of a high specificity of CLL-associated mCAs for circulating B-cell clones even stronger because it suggests that even in the cohort of individuals where the prior probability of having clonal events is higher (i.e. in the setting of cytopenias or cytoses), CLL-associated mCAs are still only rarely seen (1/104) if circulating B-cell clones are not detected on flow cytometry.*

DNA genotyping of the Mayo Clinic participants

DNA was extracted from whole blood for most individuals (100% of individuals without MBL, 99.7% LC-MBL, 90% HC-MBL, 99% SLL, and 95% CLL) and from PBMCs for the remaining individuals. *The frequency of mCAs described in this study were not statistically significantly different between individuals who had DNA extracted from whole blood versus PBMCs (data not shown).* DNA was extracted from the same blood draw as that used for flow cytometry screening among the participants in the Mayo Clinic MBL Biobank, enabling an assessment of the relationship between mCAs and MBL at the same time point. Genotyping was performed using the Infinium OmniExpress Array or the Infinium Global Screening Array. Extensive quality control was implemented as detailed elsewhere(3, 9) . The quality-controlled genotyping data was used to compute a CLL-polygenic risk score (CLL-PRS) from the weighted average of 41 SNPs previously associated with CLL(3, 9) and to estimate genetic ancestry using ADMIXTURE(10).

mCA detection from SNP-array data

Genotype intensity data was used to call mCA events with the MoChA algorithm(8), which enables detection of mCAs down to a sensitivity of 0.5-1% cell fraction. *Comparison of mCA calls from MoChA against chromosomal abnormalities identified by clinical FISH assays (in the subset of individuals from the Mayo Clinic CLL Database who had such data) revealed a high specificity (>96% across each locus) but variable sensitivity (ranging from 60% for 13q to 94% for 11q deletions) (****Supplemental Table 2****). Manual review of probe intensity measurements from SNP-array data recovered additional events and identified CNN-LOH events among those with del 13q detected by FISH. Among 260 del 13q events detected by FISH, 155 were detected as del 13q by MoChA in the post-quality control and filtered data set. Among the remaining 105, a copy-number-neutral loss of heterozygosity (CNN-LOH) was detected in 17 individuals and calls generated by MoChA prior to filtering (which could have resulted in loss of events filtered as germline events in individuals who may have homozygous deletions at this locus) identified del 13q in 29 additional individuals. In the remaining 59 individuals, manual review of B-allele fraction (BAF) and log R ratio (LRR) data was undertaken in 27 individuals. This recovered 12 additional individuals with del 13q. The fact that we were able to recover events at the 13q locus among the pre-filtered calls and by manual review of SNP probe intensity data is likely explained by the following. The frequency of individuals with MBL, CLL/SLL in our overall cohort is substantially higher than that in biobanks (where individuals are not selected for the presence of MBL, CLL, or SLL) for which the MoChA algorithm was developed. Therefore, the prior probability of finding mCAs at canonical CLL loci (including 13q) would be lower in most biobanks sampled from the general population such as the UK Biobank(11) compared to cohorts like ours. We expect that the sensitivity estimates from comparison with FISH data for the canonical CLL loci are underestimates for the rest of the genome where we would not expect as significant a difference in the prior probability of finding chromosomal alterations between our study and in other biobanks.*

Classification of mCAs

We categorized mCA events using the following definitions:

Canonical CLL-associated mCAs were defined as abnormalities typically found among CLL patients and included the following: del 6q, del 11q, trisomy 12, del 13q, and del 17p and copy-number neutral LOH events at 13q/ *MIR16-1*.

CLL driver mCAs were defined as either a canonical CLL-associated mCA event and/or those that fully contain one of the candidate driver chromosomal abnormalities from two recent, large-scale genomic sequencing studies of CLL(12, 13), which identified a union of 179 unique candidate driver chromosomal abnormalities. *As a sensitivity analysis, a more liberal classification was performed whereby a given mCA was considered a CLL driver mCA if it was either a canonical CLL-associated mCA or had any degree of overlap with at least one candidate driver chromosomal abnormality (of the right type – deletion, duplication, or copy-number neutral loss of heterozygosity). On a per-individual level, the results were highly concordant, and the results from the more conservative annotation are presented in this paper.*

Lymphoid mCAs were identified based on a pre-determined list of mCAs defined in our earlier work(14) to be specifically associated with prevalent lymphoid malignancies in the UK Biobank. *Of note, while this classification is consistent with the definition of lymphoid mCAs in our earlier work(14), it does allow for a given mCA to be a ‘CLL driver mCA’ while not meeting criteria for being a ‘lymphoid mCA’ if that mCA is also associated with myeloid malignancies in addition to being a candidate CLL driver (e.g., del 5q(12)). The results from the mCA classification are shown in* ***Supplemental Table 1****.*

*We analyzed autosomal mCAs that were not drivers of CLL or other lymphoid malignancies on a per-individual basis (rather than on a per-mCA level) to avoid possible confounding by driver mCAs (CLL driver mCA or lymphoid mCA) that may co-occur in individuals who also have a non-driver autosomal mCA.*

Statistical Analysis of mCA association with MBL, CLL, and SLL

We used logistic regression to estimate odds ratios (OR) and 95% confidence intervals (CI). We evaluated associations between the presence of (at least one mCA of) each mCA category defined above as a predictor variable and the phenotypes being compared (e.g., HC-MBL vs. LC-MBL) as the outcome variable. *Regression models were adjusted for age and/or age^2 (depending on association with phenotype in univariate analyses), sex (except when analyzing association with Loss-of-Y chromosome, which was done only in males), genetically determined European ancestry (encoded as a binary variable), DNA source (whole blood vs. PBMC), and mCA calling batch (encoded as categorial variables). Array type (Infinium OmniExpress Array or the Infinium Global Screening Array) was included in regression models in all analyses except when comparing CLL vs. SLL (given that all individuals were genotyped on the same array). Note that the sample size denominators in the forest plots (****Figure 1C, D*** *and* ***Supplemental Figure 6****) may be slightly lower than the overall sample size for each phenotype due to missing data for covariates in some individuals. In* ***Supplemental Figure 6D****, the number of autosomes on which mCAs were found per individual was analyzed as opposed to the number of mCAs per individual as a conservative measure to avoid double counting large mCA events that may have been called as a series of multiple, contiguous mCAs by MoChA.*

Ten-fold cross-validation analyses

*Regression models incorporating mCA, CLL-PRS, and absolute lymphocyte count (ALC) data were generated to evaluate classification of HC-MBL against the combined group of individuals with LC-MBL and individuals without MBL. The performance of the prediction models was estimated by using the ten-fold cross-validation approach. The baseline model included demographics (age as a continuous variable, age^2, sex). Combinations of additional predictors (mCA, CLL-PRS, ALC) were added to the baseline model to estimate the predictive power of these variables. ALC data were utilized regardless of the date of ALC ascertainment in comparison to date of flow cytometry screening because sensitivity analyses revealed similar results based on the smaller set of individuals who had ALC data within a year of flow cytometry screening. Note that the analysis in* ***Figure 4*** *and* ***Supplemental Figures 9A-C*** *were restricted to individuals with HC-MBL from the Mayo Clinic Biobank for greater generalizability to other biobanks that have sampled individuals from the general population (and to avoid inflating the predictive power of ALC for HC-MBL by inclusion of individuals with HC-MBL ascertained in clinic). The combined group of individuals with LC-MBL or without MBL was chosen as the comparator for individuals with HC-MBL to avoid inflating the performance of the predictive variables (since the predictor variables cannot discriminate LC-MBL well from individuals without MBL).*

**A**

**B**

**C**

**D**

**E**

**Supplemental Figure 1. Demographic, flow cytometric, and laboratory data for study participants from the Mayo Clinic MBL Biobank and Mayo Clinic CLL Database**. Age at the time of DNA sampling for SNP array analysis, with horizontal white bars indicating median values (**A**). Self-reported sex (**B**). Immunophenotype and number of clones detected by eight-color flow cytometry in biobank individuals, with CLL-like clones characterized by CD20 dim+, CD5+; atypical, CD2+, CD5+; non-CLL-like, CD20+, CD5-. The inset shows the total number of clones (of any immunophenotype) present (**C**). Genetic ancestry determined using ADMIXTURE(10) (**D**). Absolute lymphocyte count (ALC) for the subset of individuals who had ALC determined on the same day as flow cytometry (Biobank participants) or diagnosis (clinic participants), with horizontal black bars indicating median values and y-axis limited to 100,000 per microliter. Data are not shown for SLL patients because only two had data for date of ALC sampling (**E**).


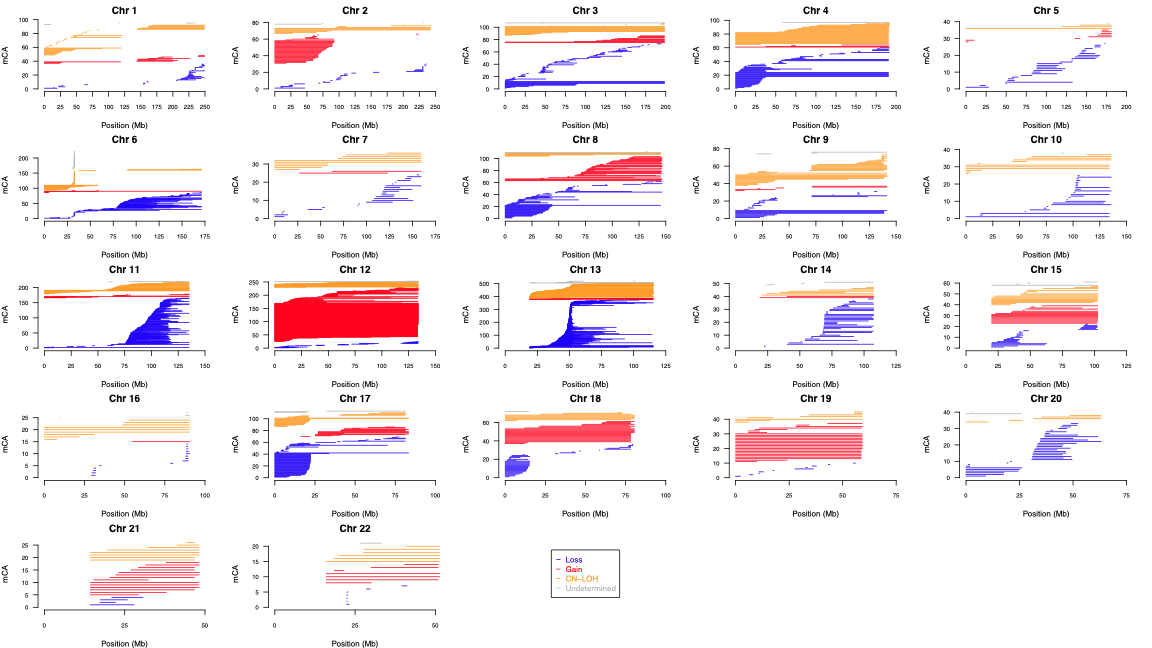


**Supplemental Figure 2**. **Distribution of autosomal mCAs among 4,632 individuals from the Mayo Clinic MBL Biobank and Mayo Clinic CLL Database**. Genomic positions are shown according to the hg19/ GRCh37 genome build. Each individual horizontal line is an mCA, with mCA type colored according to the legend on the bottom right.

**Supplemental Figure 3**. **Frequency of canonical CLL-associated mCAs and CLL driver mCAs across the spectrum of B-cell clonality**. Frequency of canonical CLL-associated mCAs (del 6q, del 11q, trisomy 12, del 13q, del 17p, copy number-neutral loss of heterozygosity at 13q/ *MIR16-1*) and CLL driver mCAs (candidate driver chromosomal abnormalities identified in Knisbacher *et al*(12) and Robbe *et al*(13); Methods).

**A**

**B**

**Supplemental Figure 4**. **Frequency of CLL-associated mCAs among HC-MBL individuals from the Mayo Clinic MBL Biobank with different immunophenotypes**. Canonical CLL-associated mCAs **(A**), CLL driver mCAs (**B**). ‘Other’ refers to presence of clones with other immunophenotypes in addition the primary one indicated.

**A**

**B**

**C**

**Supplemental Figure 5**. **Proportion of individuals with at least one mCA of the indicated category.** Lymphoid mCAs (**A**), lymphoid mCAs without known CLL driver mCAs (**B**), and autosomal mCAs without known CLL driver mCAs or lymphoid mCAs (**C**) are shown.

**A**

**B**

**C**

**D**

**E**

**F**

**Supplemental Figure 6. Association of mCAs with various states of circulating B-cell clonality**. Data are shown for CLL vs. controls with respect to presence of the specified mCAs (**A**), SLL vs. controls with respect to presence of the specified mCAs (**B**), CLL vs. HC-MBL with respect to presence of the specified mCAs **(C),** CLL vs. HC-MBL with respect to the number of autosomes on which the specified mCAs were present (**D**) as a conservative proxy for the number of mCAs present (Supplemental Methods), CLL vs. HC-MBL with respect to the cell fraction of the specified mCAs (**E**) and CLL vs. SLL with respect to the presence of the specified mCAs (**F**). In panels containing data for CLL patients (A, C, D, E, and F), the data are displayed for CLL Rai stage 0 (light purple; bottom line) and stages I-IV (dark purple; top line) separately for each category of mCA. In (A) and (B), data for individual canonical CLL-associated mCAs are not shown given their rarity among controls (and thus imprecision of effect size estimates). Data for individual canonical CLL-associated mCAs or mosaic loss of Y chromosome are not shown in (D) as the parameter analyzed here (number of autosomes) only applies to categories of mCA and not individual mCA events. The sample sizes shown may be smaller than the total number of participants within each category (e.g., HC-MBL, CLL, SLL) analyzed in our study as the sample size calculation here is based only on individuals with data available across all covariates.

**A**

**B**

**C**

**D**

**Supplemental Figure 7**. **Distribution of mCA cell fraction and number**. Distribution of the cell fraction of all mCAs detected in the Mayo cohort (**A**), CLL driver mCAs (**B**), and autosomal mCAs in individuals without a CLL driver mCA or lymphoid mCA (**C**). Distribution of the number of autosomes on which CLL driver mCAs were detected per individual (**D**).

**A**

**B**

**C**

**Supplemental Figure 8**. **Comparison of mCA cell fraction against B-cell fraction from flow cytometry among individuals in the Mayo Clinic MBL Biobank with DNA extracted from whole blood**. Data from individuals with HC-MBL, LC-MBL, or controls in the Mayo Clinic MBL Biobank whose DNA was extracted from whole blood are shown for canonical CLL-associated mCAs (**A**), CLL driver mCAs (**B**), and lymphoid mCAs (**C**). Sampling for flow cytometry and DNA extraction was done on the same date. Data points above the dashed red line indicate individuals in whom the fraction of cells containing an mCA exceeds the B-cell fraction, suggesting presence of the mCA beyond the B-cell lineage and origin prior to B-cell lineage commitment.

**A**

**B**

**C**

**D**

**Supplemental Figure 9. Test characteristics for distinguishing individuals with HC-MBL from the combined group of individuals with LC-MBL or without MBL, and from stage 0 CLL.** Area under the receiver operating characteristic curves (AUC) was calculated from ten-fold cross-validation analyses. The mCA parameter modeled was the presence of at least one canonical CLL-associated mCA (**A**), lymphoid mCA (**B**), or autosomal mCA (**C**) for distinguishing HC-MBL cases in the Mayo Clinic MBL Biobank from the combined group of individuals with LC-MBL or without MBL. This analysis is based on individuals with available data across all the predictors among HC-MBL cases in the biobank (n = 60), controls (n = 2,740), and LC-MBL (n = 669). In (**D**), the mCA parameter modeled was the presence of at least one CLL driver mCA and the test characteristics for distinguishing HC-MBL clinic cases (n = 200) from stage 0 CLL cases (n = 196) are shown. Demographics refers to age, age^2^ and sex. ALC, absolute lymphocyte count. PRS, polygenic risk score associated with CLL.

**References**

1. Shanafelt TD, Kay NE, Rabe KG, Call TG, Zent CS, Maddocks K, et al. Brief report: natural history of individuals with clinically recognized monoclonal B-cell lymphocytosis compared with patients with Rai 0 chronic lymphocytic leukemia. J Clin Oncol. 2009;27(24):3959-63.

2. Slager SL, Lanasa MC, Marti GE, Achenbach SJ, Camp NJ, Abbasi F, et al. Natural history of monoclonal B-cell lymphocytosis among relatives in CLL families. Blood. 2021;137(15):2046-56.

3. Kleinstern G, Weinberg JB, Parikh SA, Braggio E, Achenbach SJ, Robinson DP, et al. Polygenic risk score and risk of monoclonal B-cell lymphocytosis in caucasians and risk of chronic lymphocytic leukemia (CLL) in African Americans. Leukemia. 2022;36(1):119-25.

4. Kleinstern G, O'Brien DR, Li X, Tian S, Kabat BF, Rabe KG, et al. Tumor mutational load predicts time to first treatment in chronic lymphocytic leukemia (CLL) and monoclonal B-cell lymphocytosis beyond the CLL international prognostic index. Am J Hematol. 2020;95:906-17.

5. Parikh SA, Rabe KG, Kay NE, Call TG, Ding W, Leis JF, et al. The CLL International Prognostic Index predicts outcomes in monoclonal B-cell lymphocytosis and Rai 0 CLL. Blood. 2021;138(2):149-59.

6. Karlson EW, Boutin NT, Hoffnagle AG, Allen NL. Building the Partners HealthCare Biobank at Partners Personalized Medicine: Informed Consent, Return of Research Results, Recruitment Lessons and Operational Considerations. J Pers Med. 2016;6(1).

7. Boutin NT, Schecter SB, Perez EF, Tchamitchian NS, Cerretani XR, Gainer VS, et al. The Evolution of a Large Biobank at Mass General Brigham. J Pers Med. 2022;12(8).

8. Loh PR, Genovese G, Handsaker RE, Finucane HK, Reshef YA, Palamara PF, et al. Insights into clonal haematopoiesis from 8,342 mosaic chromosomal alterations. Nature. 2018;559(7714):350-5.

9. Kleinstern G, Camp NJ, Goldin LR, Vachon CM, Vajdic CM, de Sanjose S, et al. Association of polygenic risk score with the risk of chronic lymphocytic leukemia and monoclonal B-cell lymphocytosis. Blood. 2018;131(23):2541-51.

10. Alexander DH, Novembre J, Lange K. Fast model-based estimation of ancestry in unrelated individuals. Genome Res. 2009;19(9):1655-64.

11. Bycroft C, Freeman C, Petkova D, Band G, Elliott LT, Sharp K, et al. The UK Biobank resource with deep phenotyping and genomic data. Nature. 2018;562(7726):203-9.

12. Knisbacher BA, Lin Z, Hahn CK, Nadeu F, Duran-Ferrer M, Stevenson KE, et al. Molecular map of chronic lymphocytic leukemia and its impact on outcome. Nat Genet. 2022;54(11):1664-74.

13. Robbe P, Ridout KE, Vavoulis DV, Dreau H, Kinnersley B, Denny N, et al. Whole-genome sequencing of chronic lymphocytic leukemia identifies subgroups with distinct biological and clinical features. Nat Genet. 2022;54(11):1675-89.

14. Niroula A, Sekar A, Murakami MA, Trinder M, Agrawal M, Wong WJ, et al. Distinction of lymphoid and myeloid clonal hematopoiesis. Nat Med. 2021;27(11):1921-7.
